# Engagement in HIV Care and Access to Cancer Treatment Among Patients With HIV-Associated Malignancies in Uganda

**DOI:** 10.1200/JGO.18.00187

**Published:** 2019-02-14

**Authors:** Daniel H. Low, Warren Phipps, Jackson Orem, Corey Casper, Rachel A Bender Ignacio

**Affiliations:** ^1^ Swedish Family Medicine Residency at Cherry Hill, Seattle, WA; ^2^ University of Washington School of Medicine, Seattle, WA; ^3^ Fred Hutchinson Cancer Research Center, Seattle, WA; ^4^ Infectious Diseases Research Institute, Seattle, WA; ^5^ Uganda Cancer Institute, Kampala, Uganda

## Abstract

**PURPOSE:**

Health system constraints limit access to HIV and cancer treatment programs in sub-Saharan Africa. Limited access and continuity of care affect morbidity and mortality of patients with cancer and HIV. We assessed barriers in the care cascade of comorbid HIV and cancer.

**PATIENTS AND METHODS:**

Structured interviews were conducted with 100 adult patients with HIV infection and new diagnoses of cancer at the Uganda Cancer Institute. Participants completed follow-up questionnaires after 1 year to assess ongoing engagement with and barriers to care.

**RESULTS:**

The median time from new-onset cancer symptoms to initiation of cancer care at the Uganda Cancer Institute was 209 days (interquartile range, 113 to 384 days). Persons previously established in HIV care waited less overall to initiate cancer care (*P* = .04). Patients established in HIV care experienced shorter times from initial symptoms to seeking of cancer care (*P* = .02) and from seeking of care to cancer diagnosis (*P* = .048). Barriers to receiving care for HIV and cancer included difficulty traveling to multiple clinics/hospitals (46%), conflicts between HIV and cancer appointments (23%), prohibitive costs (21%), and difficulty adhering to medications (15%). Reporting of any barriers to care was associated with premature discontinuation of cancer treatment (*P* = .003).

**CONCLUSION:**

Patients with HIV-associated malignancies reported multiple barriers to receiving care for both conditions, although knowledge of HIV status and engagement in HIV care before presentation with malignancy reduced subsequent time to the start of cancer treatment. This study provides evidence to support creation and evaluation of integrated HIV and cancer care models.

## INTRODUCTION

Health system constraints often limit access to HIV treatment programs throughout sub-Saharan Africa (SSA).^1^ Specifically, transportation,^2,3^ cost,^3,4^ and availability of care^5^ present significant barriers to access and continuation of HIV care. These challenges are further magnified with comorbid conditions, such as HIV-associated malignancy (HIVAM).^6^ Understanding how barriers to care affect patients with HIVAM is important in SSA as the incidence of HIVAM continues to grow. Following the trends in the United States and Europe,^7,8^ cancer in SSA, including AIDS-defining cancer (ADC) and non-ADC (NADC), is becoming one of the leading causes of death in persons living with HIV (PLWH).^9^

In Uganda, approximately 29% of all patients with cancer are HIV positive, despite a general population prevalence of 7.5%,^10^ and the risk of death resulting from cancer is more than two-fold higher in patients with cancer who are HIV infected.^11^ Factors that may contribute to poor outcomes in patients with HIVAM include late stage at presentation and inability to obtain or negotiate complex treatments.^12^ We believe these factors may be even more challenging in settings where HIV and cancer care are not integrated. Identifying where along the care cascade delays or barriers exist may help reduce morbidity and mortality.^13^ Some of this work has already begun in oncology care in Botswana,^14^ and we aimed in this study to further explore the care cascade of comorbid HIV and cancer to more specifically identify ways in which integrated cancer and HIV care could reduce barriers. We therefore created this observational cohort of patients with HIVAM at the Uganda Cancer Institute (UCI), the sole cancer referral center in the country at the time of this study (an additional cancer center has since been established in Mbarara).

## PATIENTS AND METHODS

Between October 2015 and January 2016, patients newly registering at the UCI with confirmed diagnoses of both cancer and HIV were referred to this study by the UCI clinician performing their intake evaluation. Patient eligibility criteria were as follows: age ≥ 18 years, HIV diagnosis at least 1 month previously (confirmed by original or photocopy of laboratory results or physician documentation in the medical record), Luganda or English speaking, and access to a mobile telephone for follow-up interview. Prospective participants were contacted directly by study staff. The enrollment target of 100 was based on feasibility with available resources. We capped enrollment of patients with Kaposi sarcoma (KS) or cervical cancer based on prevalence of these cancers at the UCI to allow sufficient representation of NADC^10^; enrollment was continuous until caps were reached. Participants completed informed consent in English or Luganda. Ethical approval was received from the Fred Hutchinson Cancer Research Center institutional review board, the National AIDS Research Committee of Uganda, and the Uganda National Commission on Science and Technology.

At study entry, participants completed a single, 60-minute, interviewer-administered structured questionnaire querying demographic and socioeconomic information, literacy,^15^ performance status (Karnofsky score),^16^ timing of HIV and cancer symptoms, diagnoses and treatment, and multiple-selection questions regarding barriers to HIV and cancer care. Timing of seeking care and diagnoses were based on patient report. When specific dates were unknown, the 15th of the known month was used for approximation. UCI medical records were used to identify date of registration at the UCI and initiation of cancer care, defined as first appointment with oncologist or equivalent cancer provider. Questions were formulated with assistance of key informants, including UCI nurses, providers, and HIV counselors, as well as HIV-infected and -uninfected patients treated in the inpatient and outpatient settings. Histologic cancer diagnosis, dates of cancer care, and CD4^+^ T-cell count (CD4 count; results from within 90 days) were abstracted from UCI medical records. Phlebotomy for CD4 count was offered to participants if no eligible CD4 result was available.

Participants were contacted by mobile telephone approximately 1 year after enrollment and asked to complete a brief telephone questionnaire regarding their HIV and cancer care. If the participant was not reached after multiple attempts, calls were placed to up to three proxies whose information had been offered by the participant. Proxies were only asked questions about the participant’s vital and functional status; no HIV- or cancer-specific questions were asked. If neither the participant nor a proxy was reached, we reviewed the last documented UCI visit from the medical records.

Data were captured in REDCap (hosted by Institute for Translational Health Sciences, Seattle, WA) and analyzed in STATA (version 13.0/14.0; STATA, College Station, TX). We evaluated time intervals between steps in the care cascade via a modified Andersen Model of Total Patient Delay,^17^ starting with the participant’s self-reported date of initial cancer symptoms and ending with initiation of cancer care. We retitled appraisal/illness delay from the Andersen Model to appraisal/behavioral delay, because our participants generally did not distinguish between when they noted symptoms from when they identified they had an illness; few health care visits are appointed in this setting, so behavioral delay (rather than scheduling delay) determined when care was first sought. Given additional challenges with clinical recognition of cancer and access to diagnostics in Uganda, we inserted a diagnostic delay–step in the model between “first receiving medical attention” and “receiving cancer diagnosis”. As such, the care cascade in our model includes experiencing initial cancer symptoms to first seeking care (appraisal/behavioral delay), first seeking care to being diagnosed with cancer (diagnostic delay), being diagnosed with cancer to being referred to cancer care (scheduling/referral delay), and being referred to cancer care to initiating cancer care via first appointment with an oncologist or equivalent cancer provider (treatment delay).

We used univariable descriptive statistics for baseline health information and intervals in the cancer care cascade. *t* tests, analyses of variance, linear regressions, and χ^2^ analyses were used to assess the relationship between HIV clinical status, antiretroviral therapy (ART) use, and care intervals.

## RESULTS

A total of 103 patients were referred to this study, of whom 101 elected to participate and were subsequently enrolled. One participant was excluded from analysis because of subsequent assessment showing her tumor was benign; 100 participants were included in this final analysis. Participants were enrolled at a median of 35 days (interquartile range [IQR], 9 to 103 days) after registration at the cancer center.

The median age at enrollment was 41 years (IQR, 33 to 48 years), and 52% of patients were women. Tumor diagnoses included KS (46%), cervical cancer (19%), breast cancer (10%), esophageal cancer (6%), head and neck cancer (5%), non-Hodgkin lymphoma (4%), vulvovaginal cancer (4%), and others (6% [other cancers included: prostate (n = 2), conjunctival squamous cell carcinoma (n = 2), penile (n = 1), and melanoma (n = 1)]; Table 1). The median Karnofsky score at enrollment was 70 (IQR, 60 to 80); the median CD4 count was 341 (IQR, 157 to 545). Ninety-six percent of participants were receiving ART at time of initial interview, although only 39% could name their medications. Half of participants had completed primary school only, and 34% percent were employed outside the home. Participants traveled a median distance of 65 km (IQR, 27 to 203 km) or a median time of 2 hours (IQR, 1 to 5 hours) from their primary residence to the UCI. Thirty-five percent reported needing to spend the night during travel to the UCI.

**TABLE 1:**
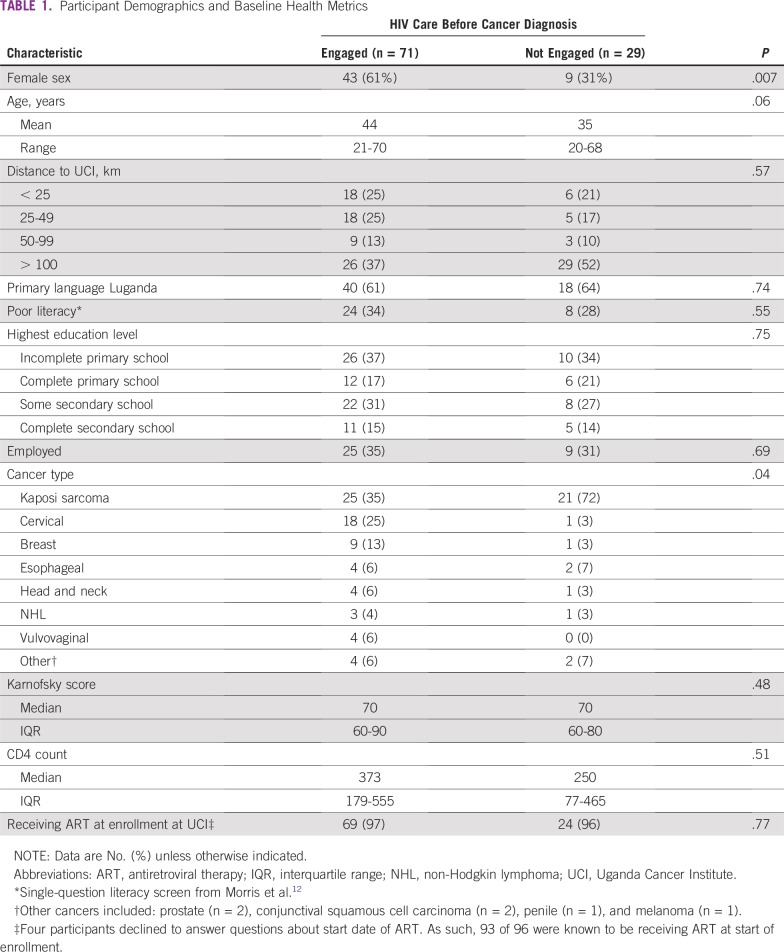
Participant Demographics and Baseline Health Metrics

**FIG 1:**
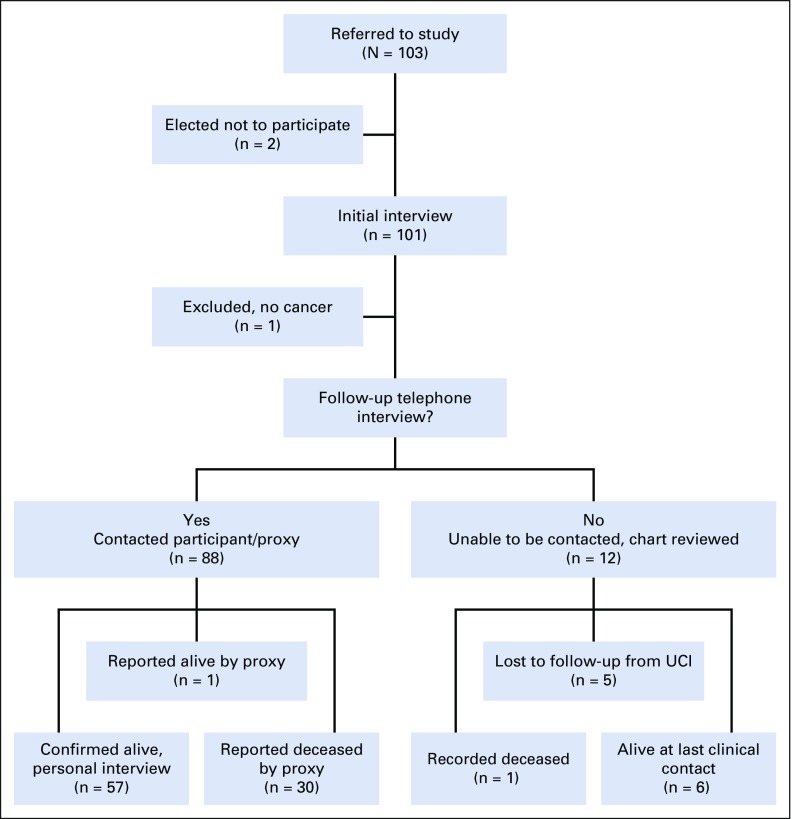
Study flow diagram of enrollment and follow-up of enrolled participants at approximately 1 year after initial interview. Loss to follow-up at the Uganda Cancer Institute was within 30 days of first clinical visit.

Eighty-eight participants or their proxies were reached for the follow-up telephone questionnaire at a median of 375 days (IQR, 369 to 379 days) after enrollment. Fifty-seven participants were confirmed alive, one was reported alive by proxy but unable to participate in the interview, and 30 were reported dead by proxy (Fig 1). Of the 12 participants/proxies not located, medical records reported last known date of clinical care at a median of 54.5 days after UCI registration (IQR, 4 to 299 days); five of these were lost to cancer care within 1 month.

### Cancer Care Cascade

No participants in this study had cancer diagnosed incidentally or by screening; all cancers were symptomatic and diagnosed before arrival to the UCI. After experiencing initial symptoms, 83% first sought treatment at a local health center or private clinic, 14% at an HIV clinic, 2% at a hospital, and 1% from an herbalist (traditional practitioner). Referrals to the UCI were generally offered on the same day a cancer diagnosis was made at an outside institution. Seventy-six percent of patients were aware of their HIV status before developing signs or symptoms of cancer; 71% had established HIV care before symptom onset. Twenty-two percent of patients were diagnosed with HIV during the course of said symptom evaluation. By arrival to the UCI, 98% of participants had been diagnosed with HIV and 96% were receiving ART. Two participants were diagnosed with HIV at the UCI. The median time of ART duration before cancer care was 489 days (IQR, 93 to 2,647 days), with 23 (24%) having started ART within 3 months (median, 30 days; IQR, 12 to 45 days) before initiating cancer care. Nineteen percent reported that their HIV providers were unaware of their cancer diagnosis.

**FIG 2:**
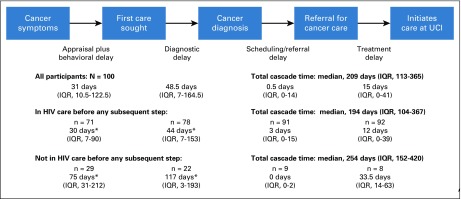
Care cascade describing time from symptomatic cancer presentation until treatment in persons with HIV-associated malignancy in Uganda. This is a modified Andersen Model of Treatment Delay, adapted for the clinical context. The first row demonstrates overall time between steps for all participants (N = 100); subsequent rows are stratified by whether the participant was receiving HIV care before entering into each step. (*) *P* < .05 between those receiving and not receiving HIV care. IQR, interquartile range; UCI, Uganda Cancer Institute.

The median time from experiencing first symptoms to initiating cancer care was 209 days (IQR, 113 to 365 days), with a median time from recognizing symptoms to first seeking medical care (appraisal/behavioral delay) of 31 days (IQR, 10.5 to 122.5 days). The median time from first seeking care to receiving a cancer diagnosis (diagnostic delay) was 48.5 days (IQR, 7 to 164.5 days). The median time from receiving a cancer diagnosis to being referred to the UCI (scheduling/referral delay) was 0.5 days (IQR, 0 to 14 days), and the median time from receiving a referral to the UCI to initiating care at the UCI (treatment delay) was 15 days (IQR, 0 to 41 days; Fig 2). Shorter time from recognizing symptoms to initiation of cancer care was associated with having previously established HIV care (*P* = .04). Having previously established HIV care reduced appraisal/behavioral delay (30 *v* 75 days for those not already receiving HIV care; *P* = .02) and diagnostic delay (44 *v* 117 days for those not receiving HIV care; *P* = .048). There was no association between engagement in HIV care and scheduling/referral or treatment delay (Fig 2). Awareness of HIV status (irrespective of HIV care engagement) was not associated with reduction in total time to initiating cancer care. Persons who were receiving ART before recognizing the symptoms determined to be associated with cancer had a total cascade duration of 207 days (IQR, 109 to 320 days), compared with those not receiving ART (318 days; IQR, 155 to 537 days; *P* = .004). Sex, age, employment, level of education, literacy, distance from participant’s home to HIV clinic, distance from participant’s home to the UCI, and cancer type were not associated with total duration within the total cascade or with any cascade interval.

### Barriers During Cancer Diagnosis and Treatment

Participants reported challenges in receiving care that affected both ART and chemotherapy adherence. Between cancer diagnosis and initiation of cancer treatment, 14 participants (15%) reported having missed ART for at least 1 week. Reported reasons for missing ART included cancer symptoms/illness limiting travel to HIV clinic (n = 9; 64%), insufficient money to travel to HIV clinic (n = 2; 14%), and other reasons not directly related to HIV or cancer (n = 3; 21%). Ninety-seven percent of participants reported they would have preferred to receive HIV care at the cancer center, an option not yet available at time of study.

Of the 57 participants who completed the follow-up interview, 38 (67%) were still receiving cancer treatment at that time, and five patients (9%) had completed all prescribed cancer treatment. Fourteen participants (25%) had prematurely stopped cancer care. All participants were still receiving HIV care and were receiving ART at time of follow-up, although only 26 (46%) could name their ART medications. Of participants interviewed at follow-up, 39 (68%) reported barriers to receiving care for their comorbid diseases, including all 14 participants no longer receiving care at the UCI. Barriers reported included travel to multiple clinics/hospitals (n = 18; 46%), conflicts between appointments for HIV and cancer care (n = 9; 23%), treatment costs (n = 8; 21%), and difficulty adhering to the quantity of medications (n = 6; 15%). Although no direct question related to stigma was asked, two participants specifically highlighted stigma as a barrier. Reporting any barrier to care at follow-up was associated with having prematurely withdrawn from cancer care (36% *v* 0%; 95% CI, 21% to 51%; relative risk not calculable; *P* = .003). Distance from place of residence to the UCI was not associated with reporting of a barrier to care; however, those who prematurely withdrew from care generally lived farther from the UCI than those who completed all prescribed cancer treatment (median distance, 172.5 *v* 40 km; *P* = .056).

## DISCUSSION

We conducted one of the first studies to our knowledge examining specific challenges faced by persons with HIVAM in obtaining diagnosis, management, and treatment of their comorbid disease. We observed that the interval from symptom recognition to initiation of cancer treatment was long for HIV-positive patients with cancer in Uganda. Regardless of whether one was engaged in HIV care before symptom recognition, the average patient waited 7 months between recognition of symptoms and initiation of treatment. This finding is concordant with prior studies of patients with KS in Uganda^18^ and both HIV-infected and -uninfected patients in Botswana,^14^ while augmenting and uniquely elaborating on the interaction between HIV care and cancer care sought by these patients.

We propose several explanations for how prior engagement in HIV care may be associated with shorter time to cancer treatment, specifically in terms of appraisal/behavioral delay and diagnostic delay. Possible patient-centered explanatory factors for the appraisal/behavioral delay include higher levels of general or health literacy, trust in allopathic medicine, less perceived stigma, more social support, or socioeconomic means in persons engaged in HIV care. Such patient-centered factors remain challenging intervention points. The diagnostic delay that we observed, however, may have been a result of systems-based factors that may be easier targets for health services interventions. The shorter observed diagnostic delay likely reflects systems-based advantages among persons engaged in HIV care, including better access to clinicians and diagnostic services. Access advantages may be less affected by patient-centered factors, including simply knowing one’s HIV status. Because we did not have a comparator arm of HIV-uninfected patients, we are uncertain to what extent observed differences were specific to engagement in HIV care, although access to other forms of longitudinal primary/preventative care is rare in most of SSA.^19^ Meanwhile, research from Rwanda and Botswana shows conflicting effects of access to traditional healers on time to oncologic care.^14,20^ Because referrals to the UCI mainly came from HIV providers and district hospitals, we believe the engagement in HIV care may have reduced diagnostic delays.

Our study findings also support efforts at improving cancer screening and diagnostics within HIV care. For example, training HIV providers in cervical cancer screening has proven beneficial and cost effective in SSA.^21,22^ Additional diagnostic delays of cancer may be reduced among HIV-infected populations by providing additional education and training on screening and diagnosis to ART providers with respect to common NADCs, such as breast, colon, lung, and head and neck cancers. Such training could further reduce time to diagnosis and treatment of these conditions that are often not considered in PLWH until all infectious causes have been ruled out.

Our study also highlights specific barriers to maintaining simultaneous treatment of HIV and cancer. Prior studies from this region have found that persons seeking HIV care often report barriers related to cost, transportation, and access to care.^23^ Our cohort similarly noted these challenges, but we also found that persons with comorbid cancer face unique barriers; conflicts between appointment times and travel between disparate and sometimes distant HIV and cancer care sites accounted for more than two thirds of challenges reported by patients. Such challenges were associated with prematurely withdrawing from cancer care. To address these challenges, cancer centers could be better positioned to care for and treat PLWH. We previously estimated that approximately one in 10 Ugandan patients with cancer, or one third of those who are HIV infected, have undiagnosed HIV^10^; therefore, cancer centers can improve efforts at universal screening and treatment of HIV. On-site provision of ART could help limit challenges of transportation, cost, and overlapping appointments, in addition to minimizing drug-drug interactions, adverse events, and gaps in ART during inpatient care or intensive outpatient treatment. Such clinics could initiate ART for patients with new HIV diagnoses, which could have benefited the 22% of persons in our study who reported that they received their HIV diagnosis within the last 3 months. Nearly all participants in our cohort felt that colocated ART during cancer treatment was not only acceptable but preferable to maintaining separate continuity HIV care elsewhere.

Our study has several limitations. We did not include a comparator arm of HIV-negative patients, because this study was designed as preparation for implementation studies in HIVAM care. Because we selected only patients who were aware of their HIV diagnosis and who ultimately initiated cancer care, our study is not representative of all persons with HIVAM, especially those with undiagnosed HIV during cancer treatment (up to 10% of patients with cancer^10^) or those who never entered cancer care. Although the UCI serves an international catchment within eastern Africa, a majority of our patients lived relatively close to Kampala, were receiving ART, were engaged in HIV care, and had access to a cellphone.^24^ We therefore acknowledge that this cohort was drawn from a population that mainly succeeded in accessing both HIV and cancer care, which is an inherent bias in recruiting from a tertiary referral center, but this was the only feasible way to identify patients with HIVAM in this setting. Our study was also limited by self-report of HIV care and events before registration at the cancer center, with attendant possibility of social desirability bias in interview responses. Recall bias may have also influenced results, because significant time had elapsed between initial presentation and study participation. Lastly, because there is no robust death registry in Uganda outside of Kampala, ascertainment of date of death was dependent on report by proxy or censoring at last care episode for patients lost to follow-up.

This study highlights opportunities to reduce delays in initiating cancer treatment in persons with HIVAM and the role that HIV care may play in improving this process. Providing care for PLWH and cancer presents many challenges, with patients most notably struggling with travel distances, cost of care, and discordant and interrupted treatment. Ultimately, coordinated HIV and cancer care could mirror the paradigm shift occurring in the management of comorbid HIV and tuberculosis (TB).^25,26^ Whereas HIV and TB programs historically functioned independently,^27^ more recently, such programs have integrated,^28^ with WHO HIV/TB collaborative guidelines,^29^ strategic frameworks,^30^ and policies.^31,32^ Just as the international community recognized that earlier integration of HIV and TB care could reduce the burden of HIV-associated TB,^33^ integration of HIV and cancer care may reduce the morbidity and mortality of comorbid HIV and cancer, while improving quality of life and reducing some barriers highlighted in our study. Strategies to improve coordination of HIV and cancer care warrant further investigation.

## Corey Casper

**Consulting or Advisory Role:** Janssen, GlaxoSmithKline, Temptime, Viracta Therapeutics

**Research Funding:** Janssen (Inst)

**Patents, Royalties, Other Intellectual Property:** Publication royalties from UpToDate

**Travel, Accommodations, Expenses:** Temptime, GlaxoSmithKline

No other potential conflicts of interest were reported.
